# Clinical Performance of OncoPredict HPV Screening Assay on Self‐Collected Vaginal and Urine Specimens Within the VALHUDES Framework

**DOI:** 10.1002/jmv.70079

**Published:** 2024-11-26

**Authors:** Chiara Giubbi, Marianna Martinelli, Ardashel Latsuzbaia, Kate Cuschieri, Hana Elasifer, Anna Daniela Iacobone, Fabio Bottari, Andrea Fausto Piana, Roberto Pietri, Giancarlo Tisi, Franco Odicino, Marc Arbyn, Clementina Elvezia Cocuzza, European VALHUDES Working Group, Federica Perdoni, Fabio Landoni, Silvia Martella, Eleonora Petra Preti, Maria Elena Guerrieri, Rita Passerini, Maria Eugenia Ghi, Maria Paola Bagella, Adriano Marrazzu, Narcisa Muresu, Illari Sechi, Arianna Dettori, Arcadia Del Rio, Ruth Chinyere Njoku, Sharon Moncur, Geraldine Breda Anthony, Sharon McPherson, Federica Salinaro, Elisa Gozzini, Franco Gargiulo, Enrico Sartori, Federico Ferrari, Arnaldo Caruso, Francesca Caccuri, Cara Martin, Ola Ibrahim, John O'Leary

**Affiliations:** ^1^ School of Medicine and Surgery University of Milano‐Bicocca Milan Italy; ^2^ Unit of Cancer Epidemiology, Belgian Cancer Centre, Sciensano Brussels Belgium; ^3^ Scottish HPV Reference Laboratory, Department of Lab Medicine Royal Infirmary of Edinburgh NHS Lothian Edinburgh UK; ^4^ HPV Research Group, Centre for Reproductive Health University of Edinburgh Edinburgh UK; ^5^ Preventive Gynecology Unit European Institute of Oncology IRCCS Milan Italy; ^6^ General Clinical Laboratory with Specialized Areas Clinical Pathology – Microbiology and Virology European Institute of Oncology IRCCS Milan Italy; ^7^ Department of Medicine, Surgery and Pharmacy University of Sassari Sassari Italy; ^8^ U.O. Coordinamento Consultori Familiari, ASSL Sassari – ATS Sardegna Sassari Italy; ^9^ Gynecologic and Obstetrical Division University of Brescia‐Spedali Civili di Brescia Brescia Italy; ^10^ Department of Human Structure and Repair, Faculty of Medicine and Health Sciences University Ghent Ghent Belgium; ^11^ Department of Biomedical Sciences University of Sassari Sassari Italy; ^12^ Department of Obsterics and Gynaecology Royal Infirmary of Edinburgh Edinburgh UK; ^13^ Department of Molecular and Translational Medicine, Institute of Microbiology University of Brescia‐Spedali Civili Brescia Italy; ^14^ Section of Microbiology, Department of Molecular and Translational Medicine University of Brescia Brescia Italy; ^15^ Department of Pathology The Coombe Women and Infants University Hospital, & Trinity College Dublin Ireland

**Keywords:** cervical cancer, diagnostic accuracy study, European VALHUDES, Human Papillomavirus, OncoPredict HPV SCR, self‐sampling

## Abstract

The introduction of self‐sampling in cervical cancer screening has raised the importance of HPV test validation on self‐collected samples. This study aimed to evaluate the clinical accuracy of the OncoPredict HPV Screening (SCR) assay on self‐collected vaginal and first‐void urine (FVU) samples, relative to cervical specimens, using the VALHUDES Framework. FVU and vaginal self‐samples followed by a clinician‐collected cervical brushing were collected from 500 women referred to colposcopy and tested using OncoPredict HPV SCR assay. The assay demonstrated clinical sensitivity to detect cervical intraepithelial neoplasia grade 2 or worse (≥ CIN2) similar to cervical samples in FVU (ratio: 0.95, [95% CI: 0.88–1.02]) and vaginal self‐samples (ratio: 0.96 [95% CI: 0.90–1.02]). The clinical specificity for < CIN2 was lower in vaginal (ratio: 0.90 [95% CI: 0.84–0.96]) but not in FVU samples (ratio: 1.03 [95% CI: 0.96–1.12) when compared to cervical samples. However, the relative specificity improved following cut‐off optimization (ratio: 0.94, 95% CI: [0.88–1.01]). Moderate to excellent agreement in HPV detection between self‐collected and cervical samples was demonstrated (Kappa values: 0.53–1.00). To conclude, OncoPredict HPV SCR assay demonstrated similar accuracy on FVU and cervical samples. On vaginal compared to cervical samples sensitivity was similar with a lower specificity, which improved with cut‐off optimization.

## 1 Introduction

Women who do not participate to cervical cancer screening programs have the highest risk of developing cervical cancer. For this reason a key target of the World Health Organization (WHO) Call to Action is to achieve the participation of at least 70% of eligible women to cervical cancer screening using a high‐performance test by the age of 35, and again by the age of 45, in support of the elimination of cervical cancer by 2030 [1]. Human Papillomavirus (HPV) testing has been demonstrated to be more effective than cytology for the secondary prevention of cervical cancer [2, 3] and is therefore recommended as a primary screening tool in current screening algorithms [4]. Additionally, meta‐analyses have shown that the clinical accuracy of PCR‐based HPV tests on self‐samples can be similar to that on cervical samples. Furthermore, offering self‐samples could increase participation of women to cervical cancer screening [5, 6].

In 2021, 48 countries worldwide recommended primary HPV‐based screening and 17 introduced self‐sampling in their national programs or guidelines [7] as response to the WHO Call to Action [1].

While several HPV tests are currently validated for use in cervical cancer screening [8] according to the VALGENT Framework and Meijer Guidelines [9, 10], only a few are formally validated for their use on self‐collected specimens. The VALidation of HUman papillomavirus assays and collection Devices for Self‐samples and urine samples (VALHUDES) Framework has defined a standardized protocol to assess the clinical performance of HPV tests in combination with self‐collection devices [11], to evaluate HPV tests previously validated on clinician‐collected cervical samples. Results of a first installment of VALHUDES demonstrated similar accuracy of first void urine (FVU) and vaginal self‐collected specimens compared to clinician‐collected cervical samples of several PCR‐based HPV assays in combination with different sample collection devices [12, 13, 14, 15, 16, 17]. This study reports on a second iteration of VALHUDES undertaken in a different geographic setting and utilizing a different approach to vaginal sampling.

The OncoPredict HPV Screening (SCR) assay, previously validated for its use in cervical cancer screening on clinician‐taken cervical samples [18], is a partial genotyping assay able to detect HPV‐16, and HPV‐18 individually and 11 “other” hrHPV genotypes (HPV‐31, ‐33, ‐35, ‐39, ‐45, ‐51, ‐52, ‐56, ‐58, ‐59, and ‐68) as a pool. Moreover, an innovative feature of this assay is that it includes an accurate sample adequacy control, particularly important in the quality assurance of testing samples which have been self‐collected. The present study aimed to evaluate the clinical performance of the OncoPredict HPV SCR assay on vaginal self‐samples collected using FLOQSwab, transported dry and subsequently resuspended in 5 mL eNAT, and on FVU, collected using the Colli‐Pee device, as compared to clinician‐collected cervical scrapes to detect high‐grade cervical lesions. Secondarily, we investigated the analytical performance of the assay and evaluated the adequacy of self‐collected samples.

## 2 Materials and Methods

### 2.1 Study Design

The European VALHUDES study was performed in compliance with ISO 20916:2019 (In vitro diagnostic medical devices—Clinical performance studies using specimens from human subjects—Good study practice) and reported in ClinicalTrials. gov Register (NCT04312737).

Within the European VALHUDES Framework, 600 women, referred to colposcopy following a previous cervical abnormality or HPV positivity, were enrolled between July 2020 and February 2022 in four colposcopy centers (NHS Lothian, Edinburgh; ASST degli Spedali Civili di Brescia, Brescia, Italy; European Institute of Oncology IRCCS, Milan, Italy; U.O. Coordinamento Consultori Familiari, ASSL Sassari ‐ ATS Sardegna, Sassari, Italy). Exclusion criteria have already been described [19].

All women were asked to self‐collect a urine sample followed by a vaginal specimen. FVU was collected using Colli‐Pee FV5000 (Novosanis, Wijnegem, Belgium). The device captures approximately 13 mL of FVU that are mixed with 7 mL nucleic acid preservative included within the collection device. Vaginal self‐collection was performed using a FLOQSwab (Copan Italia Spa, Brescia, Italy). During gynecological examination, a cervical specimen was collected by a clinician with Cervex‐Brush (Rovers Medical Devices, Oss, The Netherlands) and immediately transferred in 20 mL PreservCyt (Hologic Inc., Bedford, Massachusetts, USA).

All women underwent colposcopy and biopsy was performed only if clinically required, according to the local protocols. The histological result of the biopsy was used to determine the disease outcome: lesions were classified as Cervical Intraepithelial Neoplasia grade 0 (CIN0), CIN1, CIN2, CIN3 and Cervical Cancer [20]. Cervical intraepithelial neoplasia grade 2 or worse (≥ CIN2) were considered as high‐grade lesions, while lesions < CIN2 were considered low‐grade lesions.

Self‐collected vaginal samples were transported dry to the laboratory together with the 20 mL PreservCyt vial containing cervical samples and the Colli‐Pee tube containing FVU. All specimens were transported to the laboratories affiliated with the enrolling colposcopy centers. After arrival in the laboratories, cervical and FVU specimens were shaken for 30 s and divided into 1.5 mL aliquots. The dry vaginal swabs were resuspended in 5 mL of PreservCyt (Hologic Inc., Bedford, Massachusetts, USA) for the first 100 women enrolled in the study; subsequently vaginal samples collected by the remaining 500 women were resuspended in 5 mL eNAT (Copan Italia Spa, Brescia, Italy). Vaginal samples were further aliquoted into 0.4 mL volumes. All aliquots were stored at −20°C until transferred to MIRRI‐IT Biobank of the University of Milano‐Bicocca where they were stored at −80°C. Results reported in this manuscript are referred to those 500 women whose vaginal swabs were resuspended in 5 mL eNAT.

### 2.2 HPV Testing

Testing of all specimens was performed at the Laboratory Clinical Microbiology and Virology, School of Medicine and Surgery, University of Milano‐Bicocca (Monza, Italy). Nucleic acid extraction was performed using a Fluent 480 (Tecan, Männedorf, Switzerland) automated platform with *Quick*‐DNA/RNA MagBead (Zymo, USA) starting from 400 µL of sample. Fluent 480 workstation was also used to set‐up the real‐time PCR plate of OncoPredict HPV SCR assay (Hiantis, Milan, Italy) according to manufacturer instructions using 10 µL of mastermix and 5 µL of sample's DNA extract. The OncoPredict HPV SCR assay, previously validated for testing on cervical scrapes in a screening setting [18], is a partial genotyping assay targeting E6 and E7 DNA sequences of 13 high‐risk human papillomavirus (hrHPV) types (HPV‐16, ‐18, ‐31, ‐33, ‐35, ‐39, ‐45, ‐51, ‐52, ‐56, ‐58, ‐59, and ‐68). The test is composed of two separate real‐time PCR reactions. A quality control reaction (QC) well which allows the assessment of nucleic acid extraction recovery, by means of an exogenous control gene target added to the sample before preanalytical processing, as well as adequacy of sample collection by the determination of human cellularity using *C‐C Motif Chemokine Receptor 5* (CCR5) gene [21]. The second reaction well is used to assess the presence of HPV16, HPV18 individually and 11 “other” hrHPV types as a pool. Both wells also include an exogenous amplification control to evaluate potential PCR inhibition. PCR was carried out using a CFX384 Touch Real‐Time PCR Detection System (Bio‐Rad, USA). All results were considered valid if HPV positivity signal was reported. In case of HPV negative result(s) samples were defined as inadequate if (i) the extraction efficiency was below 10%; (ii) less than 400 cells/reaction in cervical samples [18] or 150 cells/reaction in urine and vaginal samples were detected and (iii) there was PCR inhibition in any of the two reaction wells.

PCR cycle threshold (Ct) interpretation was performed with the support of Hiantis' Reader, an Artificial Intelligence‐based reading software (CE Marked), developed for Hiantis by HiFuture, Teoresi Group Company (Italy).

Manufacturer's cut‐off for all hrHPV types in cervical samples and vaginal self‐samples was Ct ≤ 40, in FVU Ct ≤ 44. New a posteriori hrHPV positivity cut‐offs for vaginal self‐samples were Ct ≤ 39 for HPV16, Ct ≤ 37 for HPV18 and Ct ≤ 38 for “other” hrHPV.

### 2.3 Statistical Analysis

Clinical sensitivity was estimated for cervical intraepithelial neoplasia grade 2 or worse (≥ CIN2) and for cervical intraepithelial neoplasia grade 3 or worse (≥ CIN3). Specificity was estimated for < CIN2 outcome or by accepting negative colposcopy as clinical endpoint when biopsy was not taken based on the colposcopist's clinical judgment. McNemar tests was used to evaluate the accuracy differences between index and comparator tests with statistical significance accepted if *p* < 0.05 or when the 95% confidence intervals excluded 1. Cohen's kappa was employed to assess HPV test concordance between self‐ and clinician‐taken samples for the entire study population and according to disease status among specimens, categorized as: poor (0.00–0.19), fair (0.20–0.39), moderate (0.40–0.59), good (0.60–0.79), and excellent (0.80–1.00). Mann‐Whitney test was used to evaluate differences in median Ct‐values and median number of cells/reaction. All statistical analyses were conducted using Stata 16.1 (Statacorp, College Station, TX, USA).

### 2.4 Ethical Approval

The European VALHUDES study (ClincalTrail. gov: NCT04312737) was conducted in accordance with the Declaration of Helsinki and approved by the central Ethics Committee of the Coordinating Centre, ASST degli Spedali Civili di Brescia, Brescia, Italy (Ethics approval number: NP 3879‐ Studio WP6‐HPVONC) on the 16th of July 2020, and subsequently by the local Ethics Committees of the other participating centers. All women signed a written informed consent form before enrollment.

Consorzio Italiano per la Ricerca in Medicina (C.I.R.M.), Milano, Italy, performed on site and remote monitoring of the study conduction, as previously described [19].

## 3 Results

### 3.1 Study Population

**Figure 1:**
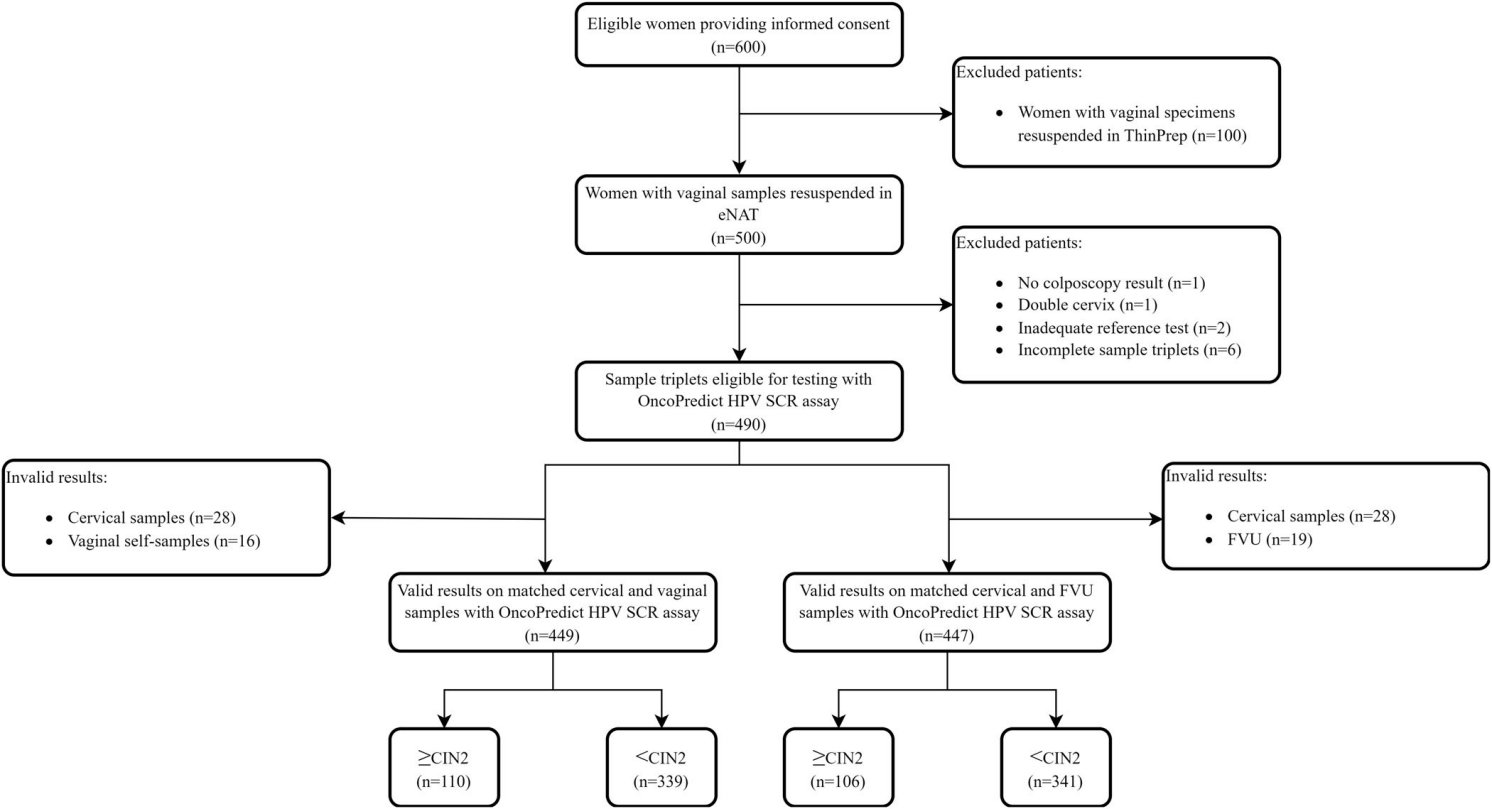
Flowchart of samples included in the analysis for the accuracy of OncoPredict HPV SCR assay within the VALHUDES Framework.

490 out of the 500 women were included in the study as reported in Figure 1. The median age of the women included in the study was 37 years (IQR: 31–47 years, range: 25–64 years). Median age of women with ≥ CIN2 lesions was significantly lower than those with < CIN2, as previously described [19]. 489 women had colposcopy with the following outcomes: 134 (27.4%) negative, 245 (50.1%) minor colposcopy findings, 104 (21.3%) major colposcopy and 6 (1.2%) suspicion of cancer. 55% (271/490) of women underwent biopsy and diagnosis of ≥ CIN2 was confirmed in 41.3% (112/271) of cases. Table 1 reports the characteristics of the study population by age group and colposcopy center. 28 cervical specimens, 16 vaginal swabs and 19 FVU samples were excluded from the analysis because they were inadequate and HPV‐negative (Figure 1). Finally, the number of matched cervical and vaginal samples available from the same woman was 449, while the number of matched cervical specimens and urine from the same woman was 447 as reported in Figure 1.

**Table 1:** Disease outcomes by biopsy and/or colposcopy findings divided by age group and colposcopy center.

Age group (years)	Participants (*n* (%))	Disease outcome		
		≥ CIN2 (*n* (%))	≥ CIN3 (*n* (%))	< CIN2 (*n* (%))*
< 30	93 (19.0)	23 (20.5)	14 (20.0)	70 (18.5)
≥ 30	397 (81.0)	89 (79.5)	56 (80.0)	308 (81.5)
Total	490 (100.0)	112 (100.0)	70 (100.0)	378 (100%)

Colposcopy center	Participants (*n* (%))	Disease outcome		
		≥ CIN2 (*n* (%))	≥ CIN3 (*n* (%))	< CIN2 (*n* (%))*
Edinburgh	191 (39.0)	37 (33.0)	27 (38.6)	154 (40.8)
Brescia	49 (10.0)	5 (4.5)	0 (0.0)	44 (11.6)
Milan	150 (30.6)	63 (56.2)	41 (58.6)	87 (23.0)
Sassari	100 (20.4)	7 (6.3)	2 (2.8)	93 (24.6)
Total	490 (100.0)	112 (100.0)	70 (100.0)	378 (100%)

Abbreviation: CIN, cervical intraepithelial neoplasia.

* 217 cases were classified as < CIN2 based on colposcopy findings without biopsy.

### 3.2 Sample's Adequacy

All hrHPV‐positive samples were considered valid. 5.7% (28/490) cervical, 3.3% (16/490) vaginal and 3.9% (19/490) FVU hrHPV‐negative specimens were inadequate. Most invalid cervical (23/28) and FVU (12/19) samples resulted from a low cellularity, while only 3 vaginal samples showed cellularity below the cut‐off. Invalidity in this group of samples was mainly related to extraction efficiency (13/16). As shown in Table 2, the cellularity of vaginal self‐collected specimens (resuspended into 5 mL of eNAT) was demonstrated to be more than 10‐fold higher than that of cervical (resuspended into 20 mL PreservCyt) and FVU samples.

**Table 2:** Median values of cellularity (cells/reaction) across different types of samples.

	Matched cervical and vaginal specimens (*n* = 449)*			Matched cervical and FVU specimens (*n* = 447)*		
	Clinician‐ collected cervical samples	Vaginal self‐collected samples	*p* value	Clinician‐collected cervical samples	FVU self‐collected samples	*p* value
**Median cellularity (IQR)**	3875 (1469–8956)	42049 (25 823–59 300)	< 0.001	3925 (1549–8902)	2879 (1220–6130)	< 0.001

* Invalid samples have been excluded from the analysis.

### 3.3 Clinical Accuracy of OncoPredict HPV SCR Assay

Clinical sensitivity for the detection of ≥ CIN2 and ≥ CIN3 and specificity for the detection of < CIN2 of OncoPredict HPV SCR assay on self‐collected samples relative to cervical scrapes are reported in Table 3. Using manufacturer's cut‐offs, the absolute sensitivity for the detection of ≥ CIN2 lesions was 87% (95% CI: 80%–93%) on cervical samples, 82% (95% CI: 74%–89%) on vaginal self‐samples and 81% (95% CI: 72%–88%) on FVU. Absolute specificity for < CIN2 detection were respectively 54% (95% CI: 49%–60%), 50% (95% CI: 44%–55%) and 57% (95% CI: 52%–62%). Absolute clinical sensitivity and specificity results are reported in Supporting Information S1: Table 1. Clinical sensitivity of OncoPredict HPV SCR assay for ≥ CIN2 on matched FVU and vaginal samples was not different to that of cervical specimens (relative sensitivity FVU/cervical sample = 0.95 [95% CI: 0.88–1.02]) (relative sensitivity vaginal self‐sample/cervical specimen = 0.96 [95% CI: 0.90–1.02]). Specificity for < CIN2 on FVU was similar to cervical scrapes (ratio = 1.03 [95% CI: 0.96–1.12]), whereas specificity on vaginal samples was slightly lower (ratio = 0.90 [95% CI: 0.84–0.96]).

**Table 3:** Relative accuracy of OncoPredict HPV SCR assay on vaginal and FVU self‐samples versus cervical specimens.

	Relative sensitivity [95% CI] for ≥ CIN2 detection	Relative sensitivity [95% CI] for ≥ CIN3 detection	Relative specificity [95% CI] for < CIN2 detection
**Manufacturer cut‐offs** a			
Vaginal self‐sample	0.96 [0.90–1.02]	0.95 [0.87–1.04]	0.90 [0.84–0.96]
FVU	0.95 [0.88–1.02]	0.93 [0.85–1.03]	1.03 [0.96–1.12]
**New cut‐offs** b			
Vaginal self‐sample	0.95 [0.90–1.00]	0.93 [0.86–1.01]	0.94 [0.88–1.01]

Abbreviations: CI, confidence interval; CIN, cervical intraepithelial neoplasia.

^a^ Manufacturer's positivity threshold for all hrHPV types in cervical samples and vaginal self‐samples: Ct ≤ 40; in FVU: Ct ≤ 44.

^b^ New a posteriori cut‐offs vaginal self‐sample: HPV16 Ct ≤ 39, HPV18 Ct ≤ 37, other hrHPV Ct ≤ 38.

After cut‐off adjustment, the relative specificity on vaginal samples improved (ratio = 0.94 [95% CI: 0.88–1.01]). Similar clinical accuracy estimates were observed when restricting the analysis for women of 30 years or older as reported in Supporting Information S1: Table 2.

### 3.4 hrHPV Positivity and Concordance

Out of 449 women with matched cervical and vaginal specimens, 256 (57.0%) cervical and 270 (60.1%) vaginal specimens were hrHPV‐positive. Out of 447 matched cervical and FVU samples, 250 (55.9%) cervical swabs and 256 (57.3%) FVU tested hrHPV‐positive.

Moderate to excellent agreement with Kappa values ranging from 0.53 to 1.00 between both vaginal and FVU self‐collected samples and cervical scrapes was demonstrated using manufacturer's cut‐offs (Tables 4 and 5). In general, vaginal samples showed higher test agreement with cervical specimens than FVU. Similar concordance rates were observed between self‐collected vaginal and clinician‐collected cervical samples using new cut‐offs as shown in Supporting Information S1: Table 3.

**Table 4:** Concordance between self‐collected vaginal and clinician‐collected cervical samples using manufacturer's cut‐offs.

	HPV type	+/+	+/−	−/+	−/−	Agreement [%]	Kappa [95% CI]
**Total population (*n*** = **449)**	hrHPV	241	15	29	164	90.2	0.798 (0.742–0.855)
	HPV16	60	7	10	372	96.2	0.854 (0.786–0.921)
	HPV18	13	2	4	430	98.7	0.806 (0.654–0.957)
	Other hrHPV	185	13	38	213	88.6	0.773 (0.714–0.831)
**≥ CIN2 (*n*** = **110)**	hrHPV	90	6	2	12	92.7	0.708 (0.519–0.897)
	HPV16	34	4	5	67	91.8	0.820 (0.708–0.933)
	HPV18	3	1	0	106	99.1	0.853 (0.568–1.000)
	Other hrHPV	64	4	5	37	91.8	0.826 (0.717–0.935)
< **CIN2 (*n*** = **339)**	hrHPV	151	9	27	152	89.4	0.788 (0.723–0.853)
	HPV16	26	3	5	305	97.7	0.854 (0.754–0.953)
	HPV18	10	1	4	324	98.5	0.792 (0.616–0.969)
	Other hrHPV	121	9	33	176	87.6	0.747 (0.677–0.818)

*Note:* Color legend: for the concordance: dark green (1.00 ≥ K > 0.80): excellent; light green (0.80 ≥ K > 0.60): good; yellow (0.60 ≥ K > 0.40): moderate; orange (0.40 ≥ K > 0.20): fair; red (0.20 ≥ K > 0.00): poor. +/+ positive on vaginal and cervical samples, +/− positive only on cervical samples, −/+ positive only on vaginal samples, −/− negative on both sample types. Manufacturer's positivity threshold for all hrHPV types in cervical samples and vaginal self‐samples Ct ≤ 40.

Abbreviations: 95% CI, 95% confidence interval; CIN, cervical intraepithelial neoplasia; N, number.

**Table 5:** Concordance between FVU and clinician‐collected cervical samples using manufacturer's cut‐offs.

	HPV type	+/+	+/−	−/+	−/−	Agreement [%]	Kappa [95% CI]
**Total population (*n*** = **447)**	hrHPV	212	38	27	170	85.5	0.707 (0.641–0.773)
	HPV16	56	10	6	375	96.4	0.854 (0.784–0.924)
	HPV18	11	3	3	430	98.7	0.779 (0.607–0.951)
	Other hrHPV	156	38	35	218	83.6	0.667 (0.597–0.737)
**≥ CIN2 (*n*** = **106)**	hrHPV	84	8	3	11	89.6	0.607 (0.398–0.816)
	HPV16	31	6	5	64	89.6	0.770 (0.642–0.898)
	HPV18	2	1	1	102	98.1	0.657 (0.212–1.000)
	Other hrHPV	56	10	5	35	85.9	0.706 (0.569–0.843)
< **CIN2 (*n*** = **341)**	hrHPV	128	30	24	159	84.2	0.681 (0.603–0.759)
	HPV16	25	4	1	311	98.5	0.901 (0.816–0.987)
	HPV18	9	2	2	328	98.8	0.812 (0.632–0.992)
	Other hrHPV	100	28	30	183	83.0	0.638 (0.554–0.723)

*Note:* Color legend: for the concordance: dark green (1.00 ≥ K > 0.80): excellent; light green (0.80 ≥ K > 0.60): good; yellow (0.60 ≥ K > 0.40): moderate; orange (0.40 ≥ K > 0.20): fair; red (0.20 ≥ K > 0.00): poor. Manufacturer's positivity threshold for all hrHPV types in cervical samples: Ct ≤ 40; FVU: Ct ≤ 44. +/+ positive on FVU and cervical samples, +/− positive only on cervical samples, −/+ positive only on FVU samples, −/− negative on both sample types.

Abbreviations: 95% CI, 95% confidence interval; CIN, cervical intraepithelial neoplasia; N, number.

In matched cervical and vaginal hrHPV‐positive samples, median Ct values were significantly lower in vaginal samples compared to cervical for 11 “other” hrHPV, but not for HPV16 or HPV18 (Supporting Information S1: Figure 1). In matched hrHPV‐positive cervical and FVU samples, median Ct values were higher in FVU as compared to cervical samples. However, the difference was not significant for HPV18 (Supporting Information S1: Figure 2).

## 4 Discussion

The introduction of self‐sampling in cervical cancer screening programs, further enhanced by the COVID19 pandemics [22], is an important instrument to reach 70% screening coverage as proposed in by the WHO Call to Action [1]. A similar clinical accuracy of PCR‐based HPV tests on self‐samples and clinician‐collected cervical scrapes has been demonstrated in previous validation studies for other assays based on the VALHUDES protocol [12, 13, 14, 15, 16, 17].

The present study demonstrated that the use of OncoPredict HPV SCR assay on self‐collected vaginal specimens using FLOQSwabs, resuspended in 5 mL of eNAT, and FVU collected using Colli‐Pee FV5000 has a similar clinical accuracy to detect ≥ CIN2+ and ≥ CIN3 lesions as compared to testing clinician‐collected cervical samples. Clinical sensitivity of OncoPredict HPV SCR assay on FVU and vaginal samples was similar to that on cervical specimens, however specificity on vaginal samples was lower when applying manufacturer cut‐off values. Cut‐off optimization on vaginal self‐collected samples resulted in an improvement in specificity without compromising sensitivity. A lower specificity for the detection of < CIN2 as compared to cervical specimen was also reported for the validation of BD Onclarity HPV test on FLOQSwabs resuspended in 3 mL of BD HPV self‐collection diluent [23]. On the other hand, in the Belgian VALHUDES, where vaginal samples were resuspended in 20 mL of PreservCyt, a posteriori cut‐offs adjustment was necessary to improve the clinical sensitivity of some of the evaluated HPV assays, but was not required for specificity [13, 14].

Different preanalytical workflows of self‐collected vaginal samples may affect the clinical accuracy of the test. Therefore, optimization and standardization of the procedures for handling and testing self‐samples are fundamental to ensure an optimal performance of the assay [24]. Presently, the VALHUDES protocol has been developed to assess the performance of HPV tests in combination with self‐collection devices [11]. In the European VALHUDES, vaginal swabs have been collected using FLOQSwabs resuspended in 5 ml of eNAT medium, while FVU were collected using a 20 mL Colli‐Pee device. eNAT is a transport medium that allows the preservation of nucleic acids, denaturation of proteins and inactivation of microbial agents. It has been previously used in combination with HPV molecular assays [25, 26]; two previous studies demonstrated a good analytical performance of FLOQSwabs self‐collected vaginal samples resuspended in 5 mL eNAT as compared to cervical samples [27, 28]. Moderate to excellent agreement in the concordance of HPV test results between vaginal and cervical specimens was also demonstrated in the present study.

Both urine and vaginal self‐collected samples are well accepted by women [29], in particular this study confirmed that FVU is a noninvasive collection method with a clinical accuracy for the detection of ≥ CIN2 lesions comparable to cervical specimens, as also previously reported for other HPV assays [16, 17].

Ensuring sample adequacy is crucial in terms of quality assurance in HPV‐based primary cervical cancer screening, particularly important when testing self‐collected samples, allowing to reduce potential false‐negative results [21, 30, 31, 32]. One of the main advantages of OncoPredict HPV SCR assay is the inclusion of a thorough quality assessment, both for the preanalytical and analytical phases. The assay allows to assess the efficiency of nucleic acid extraction and potential PCR inhibition through the use of external calibrators, as well as evaluating the adequacy of sample collection through a quantitative cellularity assessment. In general, most of the clinically validated molecular assays include an internal housekeeping gene which is used for both sample adequacy and amplification assessment. Recent studies have underlined the importance of quality controls in molecular diagnostics, such as in HPV‐based primary screening, including the possibility of a quantitative sample adequacy control in a separate reaction well [30, 31]. In the present study, no invalid result related to PCR inhibition was detected, underlying the good performance of the analytical process. The invalidity due to a low extraction efficiency could be attributed to errors in specific nucleic acid extraction runs that may be resolved by retesting the sample following repeated nucleic acids extraction. On the contrary, in case of low cellularity samples, in absence of other invalidity reasons, sample collection should be repeated [30]. In general, in this study the invalidity rate was higher in cervical samples than in self‐collected samples. This could be related to different limits of acceptable cellularity for cervical and self‐collected samples. Moreover, as previously discussed, in the present study vaginal samples were resuspended in 5 mL of eNAT while cervical swabs in 20 ml of PreservCyt which may have resulted in lower sample cellularity. In conclusion, the inclusion of the QC module in OncoPredict HPV SCR assay, allowing assessment of sample adequacy, may improve confidence in the reporting of negative HPV results in cervical cancer screening.

OncoPredict HPV SCR assay is a limited genotyping assay, identifying HPV16, HPV18 and/or 11 “other” hrHPV genotypes, whereas OncoPredict HPV Quantitative Typing (QT) is a full genotyping assay that can distinguish all the 12 hrHPV types separately, based on the same preanalytical workflow. Both assays have been independently validated on cervical and self‐samples within VALGENT and VALHUDES Frameworks [18, 19, 33], respectively. In future strategies for the molecular triage of HPV‐positive self‐collected samples, OncoPredict HPV SCR screen‐positive samples may benefit from reflex testing using the quantitative, full‐genotyping OncoPredict HPV QT assay, on the same sample's nucleic acid extract.

In conclusion, following a posteriori cut‐offs adjustment the OncoPredict HPV SCR assay demonstrated similar clinical accuracy for ≥ CIN2 lesions on self‐collected vaginal and FVU samples compared to testing on clinician‐collected cervical samples.

## Author Contributions

Principal investigator and conceptualization: C.E.C., M.A. Protocol development: M.A., C.E.C. Funding acquisition: C.E.C., M.A. Project administration: C.E.C., M.A. Enrollment of patients: A.D.I., R.P. Data curation and formal analysis: A.L., M.A., M.M. Sample collection, handling and Methodology: A.D.I., R.P., M.M., C.G., F.B., A.F.P. Drafting original manuscript: C.G. Critical review and editing of manuscript: A.L., A.D.I., A.F.P., C.E.C., C.G., F.B., F.O., G.T., H.E., K.C., M.A., M.M., R.P.

## European Valhudes Working Group

Federica Perdoni, Fabio Landoni (School of Medicine and Surgery, University of Milano‐Bicocca, Milan, Italy); Silvia Martella, Eleonora Petra Preti, Maria Elena Guerrieri (Preventive Gynecology Unit, European Institute of Oncology IRCCS, Milan, Italy); Rita Passerini (General Clinical Laboratory with Specialized Areas Clinical Pathology – Microbiology and Virology, European Institute of Oncology IRCCS, Milan, Italy); Maria Eugenia Ghi, Maria Paola Bagella, Adriano Marrazzu (U.O. Coordinamento Consultori Familiari, ASSL Sassari – ATS Sardegna, Sassari, Italy); Narcisa Muresu, Illari Sechi, Arianna Dettori, Arcadia Del Rio (Department of Medicine, Surgery and Pharmacy, University of Sassari, Sassari, Italy); Ruth Chinyere Njoku (Department of Biomedical Sciences, University of Sassari, Sassari, Italy) Sharon Moncur (HPV Research Group, Centre for Reproductive Health, University of Edinburgh, Edinburgh, UK); Geraldine Breda Anthony, Sharon McPherson (Department of Obsterics and Gynaecology, Royal Infirmary of Edinburgh, Edinburgh UK); Federica Salinaro, Elisa Gozzini (Gynecologic and Obstetrical Division, University of Brescia‐Spedali Civili di Brescia, Brescia, Italy); Franco Gargiulo (Department of Molecular and Translational Medicine, Institute of Microbiology, University of Brescia‐Spedali Civili, Brescia, Italy); Enrico Sartori, Federico Ferrari (Gynecologic and Obstetrical Division, University of Brescia‐Spedali Civili di Brescia, Brescia, Italy); Arnaldo Caruso, Francesca Caccuri (Section of Microbiology, Department of Molecular and Translational Medicine, University of Brescia, Italy); Cara Martin, Ola Ibrahim, John O'Leary (Department of Pathology, the Coombe Women and Infants University Hospital, & Trinity College, Dublin, Ireland).

## Ethics Statement

The European VALHUDES study (ClincalTrail. gov: NCT04312737) was conducted under the Declaration of Helsinki. The study was approved by the central Ethics Committee of the Coordinating Centre, ASST degli Spedali Civili di Brescia, Brescia, Italy (Ethics approval number: NP 3879‐Studio WP6‐HPVONC) and subsequently by the local Ethics Committees of the other participating centers. All women signed a written informed consent form before to enrollment.

## Conflicts of Interest

The European VALHUDES is a researcher‐induced study, coordinated by the University of Milano‐Bicocca (Milan, Italy), Sciensano (Bruxelles, Belgium), Istituto Europeo di Oncologia (Milan, Italy), University of Sassari (Sassari, Italy), U.O. Coordinamento Consultori Familiari, ASSL Sassari–ATS Sardegna (Sassari, Italy), NHS Lothian, University of Edinburgh (Edinburgh, Scotland), Trinity College Dublin (Dublin, Ireland).

Manufacturers of HPV assays (GeneFirst, Oxford, UK and Hiantis, Milan, Italy) and devices (Copan Italia Spa, Brescia, Italy and Novosanis, Belgium) participated in the European VALHUDES framework under the condition of accepting independent publication of results. Funding received from the companies are managed by the directors of the respective collaborating institutions. The study group received free self‐sample collection devices from Copan Italia Spa (Brescia, Italy) and Novosanis (Belgium) and free OncoPredict HPV assay from (Hiantis, Milan, Italy).

C.E.C. declares to have received research support from BD Diagnostics, Seegene, Arrows Diagnostics, Copan, GeneFirst, Hiantis and VITRO. CEC is a minority shareholder of Hiantis.

Sciensano authors do not have any personal or material conflict of interest. A.D.I., A.F.P., F.B., F.O., G.T., H.E., K.C., M.M., C.G., R.P. declare no conflict of interest.

## Supporting information

Supporting information.

## Data Availability

Final study data sets generated by the study will be stored locally and securely at Sciensano. Anonymized data will be available by request to the corresponding author on a case‐by‐case basis pending approval from the information security coordinator at Sciensano.
