# Supplementary material for: Clinical Performance of OncoPredict HPV Screening Assay on Self‐Collected Vaginal and Urine Specimens Within the VALHUDES Framework
Source: J Med Virol. 2024 Nov 26;96(11):e70079. doi: 10.1002/jmv.70079 (PMC11590042; doi:10.1002/jmv.70079)
Supplement: Supplementary file 1 — Supporting information. [file JMV-96-e70079-s001.docx]

**Supplementary Materials**

Supplementary Table 1: Absolute sensitivity and specificity of OncoPredict HPV SCR assay on cervical, vaginal and FVU samples.

|  | **N** | **Sensitivity [95%CI] for ≥CIN2 detection** | **N** | **Sensitivity [95%CI] for ≥CIN3 detection** | **N** | **Specificity [95%CI] for <CIN2 detection** |
| --- | --- | --- | --- | --- | --- | --- |
| **Cervical samples** | 96/110 | 87% [80%-93%] | 61/69 | 88% [78%-95%] | 191/352 | 54% [49%-60%] |
| **FVU** | 87/108 | 81% [72%-88%] | 56/69 | 81% [70%-90%] | 208/363 | 57% [52%-62%] |
| **Vaginal self-samples** | 92/112 | 82% [74%-89%] | 58/70 | 83% [72%-91%] | 180/362 | 50% [44%-55%] |

CI, confidence interval; CIN, cervical intraepithelial neoplasia; FVU, first-void urine

Supplementary Table 2: Clinical accuracy of OncoPredict HPV SCR assay on vaginal and FVU self-samples versus cervical specimens in women **≥** 30.

|  | **Relative sensitivity [95%CI] for ≥CIN2 detection** | **Relative sensitivity [95%CI] for ≥CIN3 detection** | **Relative specificity [95%CI] for <CIN2**  **detection** | |
| --- | --- | --- | --- | --- |
| **Manufacturer cut-offs**^1^ | |  |  | |
| Vaginal self-samples | 0.96 [0.89-1.03] | 0.94 [0.84-1.05] | 0.92 [0.85-0.99] |  |
| FVU | 0.96 [0.88-1.04] | 0.93 [0.83-1.05] | 1.03 [0.95-1.12] |  |
| **New cut-offs**^2^ | |  |  | |
| Vaginal self-samples | 0.96 [0.89-1.03] | 0.95 [0.85-1.05] | 0.93 [0.87-0.99] |  |

CI, confidence interval; CIN, cervical intraepithelial neoplasia; FVU, first-void urine

^1^ Manufacturer’s positivity threshold for all hrHPV types in cervical samples and vaginal self-samples: Ct ≤ 40; in FVU: Ct ≤ 44.

^2^ New a posteriori cut-offs vaginal self-samples: HPV16 Ct ≤ 39, HPV18 Ct ≤ 37, other hrHPV Ct ≤ 38.

Supplementary Table 3: Concordance between self-collected vaginal and clinician-collected cervical samples using new cut-offs.

| **Total population (n=449)** | **HPV type** | **+/+** | **+/-** | **-/+** | **-/-** | **Agreement [%]** | **Kappa [95% CI]** |
| --- | --- | --- | --- | --- | --- | --- | --- |
|  | hrHPV | 237 | 19 | 25 | 168 | 90.2 | 0.799 (0.743 - 0.856) |
|  | HPV16 | 59 | 8 | 9 | 373 | 96.2 | 0.852 (0.783 - 0.921) |
|  | HPV18 | 13 | 2 | 2 | 432 | 99.1 | 0.862 (0.729 - 0.996) |
|  | Other hrHPV | 180 | 18 | 35 | 216 | 88.2 | 0.763 (0.703 - 0.823) |
| **≥CIN2 (n=110)** | **HPV type** | **+/+** | **+/-** | **-/+** | **-/-** | **Agreement [%]** | **Kappa [95% CI]** |
|  | hrHPV | 90 | 6 | 1 | 13 | 93.6 | 0.751 (0.578 - 0.925) |
|  | HPV16 | 33 | 5 | 5 | 67 | 90.9 | 0.799 (0.680 - 0.918) |
|  | HPV18 | 3 | 1 | 0 | 106 | 99.1 | 0.853 (0.568 - 1.000) |
|  | Other hrHPV | 64 | 4 | 4 | 38 | 92.7 | 0.846 (0.743 - 0.949) |
| **<CIN2 (n=339)** | **HPV type** | **+/+** | **+/-** | **-/+** | **-/-** | **Agreement [%]** | **Kappa [95% CI]** |
|  | hrHPV | 155 | 13 | 24 | 147 | 89.1 | 0.782 (0.716 - 0.848) |
|  | HPV16 | 26 | 3 | 4 | 306 | 97.9 | 0.870 (0.775 - 0.965) |
|  | HPV18 | 10 | 1 | 2 | 326 | 99.1 | 0.865 (0.714 - 1.000) |
|  | Other hrHPV | 116 | 14 | 31 | 178 | 86.7 | 0.726 (0.652 - 0.800) |

N: number; CI: 95% confidence interval; CIN: cervical intraepithelial neoplasia

+/+ positive on vaginal and cervical samples, +/- positive only on cervical samples, -/+ positive only on vaginal samples, -/- negative on both sample types.

Note: Color legend: for the concordance: dark green (1.00 ≥ K > 0.80): excellent; light green (0.80 ≥ K > 0.60): good; yellow (0.60 ≥ K > 0.40): moderate; orange (0.40 ≥ K > 0.20): fair; red (0.20 ≥ K > 0.00): poor.

Manufacturer’s positivity threshold for all hrHPV types in cervical samples: Ct ≤ 40.

New a posteriori cut-offs vaginal self-samples: HPV16 Ct ≤ 39, HPV18 Ct ≤ 37, other hrHPV Ct ≤ 38.

Supplementary Figure 1: Difference in Ct values of cervical and vaginal self-collected samples.

Supplementary Figure 2: Difference in Ct values of cervical and FVU samples.
